# Olfactory and trigeminal interaction of menthol and nicotine in humans

**DOI:** 10.1007/s00221-012-3063-2

**Published:** 2012-03-21

**Authors:** Bertold Renner, Karen Schreiber

**Affiliations:** Institute of Experimental and Clinical Pharmacology and Toxicology, University of Erlangen-Nürnberg, Erlangen, Germany

**Keywords:** Pain, Nasal irritation, Sensitization, Transient receptor potential channel, Nicotinic acetylcholine receptor

## Abstract

The purpose of the study was to investigate the interactions between two stimuli—menthol and nicotine—both of which activate the olfactory and the trigeminal system. More specifically, we wanted to know whether menthol at different concentrations modulates the perception of burning and stinging pain induced by nicotine stimuli in the human nose. The study followed an eightfold randomized, double-blind, cross-over design including 20 participants. Thirty phasic nicotine stimuli at one of the two concentrations (99 and 134 ng/mL) were applied during the entire experiment every 1.5 min for 1 s; tonic menthol stimulation at one of the three concentrations (0.8, 1.5 and 3.4 μg/mL) or no-menthol (placebo control conditions) was introduced after the 15th nicotine stimulus. The perceived intensities of nicotine’s burning and stinging pain sensations, as well as perceived intensities of menthol’s odor, cooling and pain sensations, were estimated using visual analog scales. Recorded estimates of stinging and burning sensations induced by nicotine initially decreased (first half of the experiment) probably due to adaptation/habituation. Tonic menthol stimulation did not change steady-state nicotine pain intensity estimates, neither for burning nor for stinging pain. Menthol-induced odor and cooling sensations were concentration dependent when combined with low-intensity nicotine stimuli. Surprisingly, this dose dependency was eliminated when combining menthol stimuli with high-intensity nicotine stimuli. There was no such nicotine effect on menthol’s pain sensation. In summary, we detected interactions caused by nicotine on menthol perception for odor and cooling but no effect was elicited by menthol on nicotine pain sensation.

## Introduction

In humans, taste and smell experiences are rarely elicited by just one chemical compound. For instance, sipping a glass of wine activates olfactory, gustatory, as well as somatosensory receptors, because food and beverages are composed of complex mixtures of flavors embedded in sophisticated matrices. In addition, recent evidence suggests that flavors do not act in isolation but rather influence each other’s perception and they can even change detection thresholds (Dalton et al. 2000; Diamond et al. 2005).

This paper focuses on the perception of chemicals in the human nose. Within the somatosensory system, interactions can occur on the same primary afferent nerve fiber through the activation of different receptor types, sometimes even by a single compound. Cross-modal interactions between the olfactory and somatosensory systems might happen at higher levels of the neuronal network (Cain and Murphy 1980; Schaefer et al. 2002), as well as in the periphery involving axon reflexes that do not require synaptical transmission (Bayliss 1901; Bouvet et al. 1987; Finger and Bottger 1993; Silver and Finger 2009). Polymodal activities caused by single-flavor compounds seem to be a normal occurrence, because finding a compound that only activates one sensory channel, for example, olfaction, can be quite a challenge (Doty et al. 1978).

It is well known that menthol activates olfactory receptors (minty smell) and the (trigeminal) somatosensory system (cooling and pain). Menthol’s stimulating properties are complex, as different sensory modalities and qualities—smell, cooling and pain—do not have the same threshold. Low concentrations just above the detection threshold activate the olfactory receptors, which results in odor sensation; medium concentrations evoke a cooling sensation in addition to the smell; and higher concentrations add a pain sensation in addition to the smell and cooling (Cliff and Green 1994; Kobal et al. 2000).

At the molecular level, it is now well established that menthol’s cooling sensation is mediated through transient receptor potential melastatin type 8 (TRPM8) channel activation (Peier et al. 2002; McKemy et al. 2002), while its pain sensation results from the activation of transient receptor potential ankyrin type 1 (TRPA1) channels.

Recently, it has been reported that nicotine at high concentrations activates TRPA1 channels (Talavera et al. 2009). It is also well known that nicotine activates other nociceptive transducers such as transient receptor potential vanilloid 1 (TRPV1) channels (Liu et al. 2004) and nicotinic acetylcholine receptors (nAChRs) (Renner et al. 1998; Thuerauf et al. 1999;Alimohammadi and Silver 2000; Thuerauf et al. 2006). Since both menthol and nicotine appear to activate TRPA1 channels in a similar manner (reversible, non-reactive), there is the possibility for TRPA1 channel interaction between these two compounds and, consequently, the modulation of perceived pain. A desensitizing effect of menthol on nicotine-induced activation of TRPA1 channels was recently observed in cultured cells overexpressing TRPA1 channels (Karashima et al. 2007; Talavera et al. 2009).

To our knowledge, the only study that has investigated the interaction between menthol and nicotine on human sensory perception was conducted by Dessirier et al. (2001), by applying both compounds to participants’ tongues. They found that the intensity of perceived irritation from nicotine was significantly diminished by pre-treatment with menthol. Since this study only covers a specific situation of potential menthol/nicotine interactions, more investigations need to be conducted to further our understanding of underlying mechanisms (Brand 2006; Kreslake and Yerger 2010).

In this present study, we examined the interaction between menthol and nicotine, which has been shown to exert multiple sensations in a concentration-dependent manner (Hummel et al. 1992; Thuerauf et al. 2000). Using an established model for the assessment of interactions between carbon dioxide (CO_2_) and menthol (Kobal et al. 2000), we here investigated the effects of stimulation with three concentrations of menthol (eliciting odor, cooling and pain sensations) on the intensity perception of nicotine stimuli presented at two concentrations (eliciting burning and stinging pain sensations). We thought that the usage of different levels of nicotine and menthol associated with different sensory qualities would provide a broader view on potential interactions and sensory outcomes and further our understanding of menthol and nicotine effects on the human chemical senses.

## Materials and methods

In this study, we applied state-of-the-art stimulation and recording technologies. Stimuli were applied by an olfactometer that enabled exact and reproducible presentation of menthol and nicotine with defined time characteristics (Kobal and Plattig 1978; Kobal 1981, 1985; Johnson and Sobel 2007). Recordings were fully computerized including monitoring of participants’ vigilance and attention (Kobal et al. 1990; Renner et al. 2007).

## Stimulation

Chemosensory nasal stimuli were applied using an olfactometer (OM4, Burghart Instruments, Wedel, Germany), which allowed the application of chemical stimuli without causing concomitant stimulation of mechano- or thermoreceptors (Kobal 1985). Thirty short (phasic) nicotine stimuli (one of the two concentrations at 99.14 ng/mL ±7 % and 133.57 ng/mL ±13 % measured by high-performance liquid chromatography [HPLC]; nicotine embedded in nitrogen) were applied to the left nostril (interstimulus interval of 1.5 min, stimulus duration of 1 s). The rise time of the stimulus concentration was below 100 ms (Thurauf et al. 1995). The olfactometer was operated with standard parameters (flow rate: 140 mL/min; humidity: ≥80 % relative humidity; temperature: 36.5 °C). The two concentrations were selected based on previous findings (Hummel et al. 1992; Thuerauf et al. 2000). Nicotine exerts smell sensations at levels just above the detection threshold. With increasing concentrations, nicotine additionally evokes a burning sensation. At even higher concentrations, nicotine elicits a distinguishable, sharp, stinging pain in addition to the odor and burning sensations. The two concentrations chosen for this study exerted smell and burning sensations at the lower concentration and a greater level of stinging sensation at the higher concentration. We believe that the burning pain is due to C-fiber activation, while the stinging pain is due to A-delta fiber activation (Thuerauf et al. 1999).

Tonic menthol stimuli were applied in the second half of the experiment after the 15th nicotine stimulus. As in the pilot study, where we used CO_2_ instead of nicotine (Kobal et al. 2000), three different concentrations of menthol were selected. The lowest concentration of 0.8 μg/mL was determined in the pilot study to be just above the olfactory detection threshold. The medium concentration of 1.5 μg/mL elicited an additional cooling sensation, and the highest concentration of 3.4 μg/mL added a ‘cutting’ pain sensation. In each of the experimental sessions, only one concentration of nicotine was combined with one concentration of menthol.

The concentrations for menthol and nicotine at the outlet of the olfactometer were routinely checked using analytical procedures previously described (Thurauf et al. 1995, 1999) as part of our quality assessment for clinical studies.

## Test substances

Optically and chemically pure (>99 % measured by HPLC) S(-) nicotine was stored in glass tubes in a nitrogen atmosphere (−20 °C) until the experiments were started (Dr. Mark, Chemisches Laboratorium, Worms, Germany). Crystalline L(−) menthol (>99 % measured by gas chromatography [GC]) was dissolved in 1,2 propanediol (≥99.5 % measured by GC) and was replaced before each experiment (Sigma Aldrich Chemie GmbH, Steinheim, Germany). Nitrogen (purity >99.9 %) and CO_2_ (purity >99.9 %) gases were used to run the olfactometer (Messer Griesheim GmbH, Krefeld, Germany).

## Psychophysical estimates and training

**Fig. 1:**
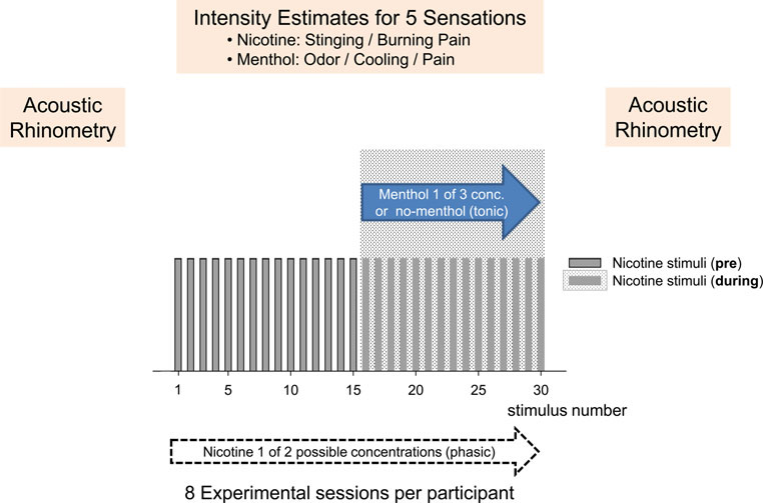
Experimental flow chart. During each experimental session, 30 phasic nicotine stimuli were applied to the nasal mucosa using an olfactometer (stimulus duration of 1 s; interstimulus interval of 1.5 min). Within one session, only one of the two nicotine concentrations was used (99 ng/mL or 134 ng/mL). In the second half of the session (i.e., after 22.5 min), the continuous background air flow was switched from clean air to one of the four menthol conditions (no menthol: placebo control; menthol-low: 0.8 μg/mL; menthol-medium: 1.5 μg/mL; menthol-high: 3.4 μg/mL). Each of the four menthol conditions was combined with one of the two nicotine concentrations. All these combinations resulted in eight experimental sessions on eight different days. Acoustic rhinometry was performed in each participant before and after each session. On the VAS, participants rated the stinging and burning pain elicited by nicotine as well as odor, cooling and pain sensations caused by tonic background menthol stimulation

We trained participants to recognize the five different sensations associated with nicotine and menthol stimulation in the nose. Nicotine stimuli used in this experiment elicited a burning and stinging pain sensation, which was different to the more cutting pain sensation elicited by higher levels of menthol. Also, the nicotine stimuli were of a short duration (1 s) compared with the sensations elicited by menthol that lasted throughout the second half of the experiment but were absent in the first half (Fig. 1). Minty odor and cooling sensations were different to the other sensations participants experienced during this study. All participants became familiar with the five different sensations and could identify each one correctly at the end of the training session.

**Fig. 2:**
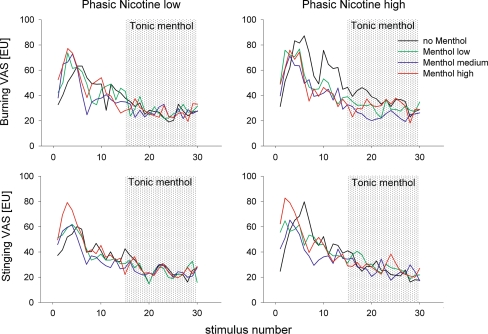
Time course of estimated mean values (19 participants) of pain intensity estimates for 30 nicotine stimuli of low (99 ng/mL) and high (134 ng/mL) concentrations that were presented throughout the entire experimental session. Interstimulus interval: 1.5 min; total duration of experiment: 45 min. During the second half of each experimental session, an additional tonic menthol background stimulus was switched on (*shadowed*) and maintained throughout the rest of the experiment. Three concentrations of menthol (menthol-low: 0.8 μg/mL, menthol-medium: 1.5 μg/mL and menthol-high: 3.4 μg/mL) and a fourth condition with clean air (no menthol) as placebo control were combined with each of the two nicotine concentrations resulting in a total of eight experiments. Participants estimated the burning pain and stinging pain sensation elicited by nicotine separately on visual analog scales (VAS) after each nicotine stimulus. After an initial sensitization, there was a uniform decline in perceived pain intensity (burning and stinging) that was not modulated by the background menthol/placebo control conditions (EU = estimation unit). The estimated mean values were derived from a linear mixed model for repeated measures using a least-square means statement

Participants were instructed to estimate both the intensity of the burning and the stinging pain after each nicotine stimulus on two separate visual analog scales (VAS) displayed on a computer screen. This resulted in a series of 30 estimates for each of the two nicotine concentrations (Figs. 1 and 2). Immediately after estimating nicotine burning and stinging intensities, participants estimated odor, cooling and pain intensities associated with menthol on a new computer display. This resulted in a series of thirty estimates for each concentration of menthol and placebo control and for each of the two nicotine concentrations used (Fig. 1). Participants did not know (a) which nicotine level or which menthol level was actually applied, (b) that concentrations for nicotine were maintained throughout the experimental session, (c) that the menthol concentration did not change once it was switched on and (d) at what time menthol was switched on.

As described above, we used two sets of VASs that were displayed as columns on a computer screen in front of the participants. They were trained to adjust the size of the columns to the level of perceived intensity by using a joystick. The initial size of a displayed column was equal to 100 estimation units (EU). At the beginning of each experimental session, the size of the column was anchored to a pain sensation elicited by CO_2_ (60 % v/v, 500 ms duration) and applied using the same olfactometer. Reducing the size of the column to nothing (0 EU) meant that no stimulus was detected. Elongating it above the initial size meant that the perceived intensity was higher than the initial anchoring sensation. The maximum length of the column was twice the original size and assigned to the value of 200 EUs. This and similar methods have been used in numerous studies carried out previously in pain research (Kobal and Hummel 1989; Kobal et al. 1990; Renner et al. 2007).

During the entire experiment, participants wore earphones through which they heard white noise (50 dB SPL) in order to cover the switching sounds coming from the olfactometer. They were also asked to perform a simple tracking task on the same computer screen once they had completed their intensity estimations. For that, they used the same joystick to keep a small red square inside a larger green one that randomly moved around on the computer screen (Kobal et al. 1990). This procedure helped to stabilize the participants’ vigilance and attention but the corresponding data were not analyzed further.

## Acoustic rhinometry

Nasal cavity geometry was assessed using acoustic rhinometry before and after each experiment. This provided information on changes in the size of the cross-section along the depth of the nasal cavity and enabled us to obtain information about potential effects of the different stimulation conditions on the volume of the nasal cavity (Rhinoklack, STIMOTRON Instruments, Wendelstein, Germany).

## Study design and study population

Each study session started at the same time, either in the morning or in the afternoon, with an assessment of participants’ health status and eligibility regarding a reduced set of exclusion and inclusion criteria (see below).

Throughout the experimental session (‘the experiment’), which lasted at least 45 min, 30 phasic nicotine stimuli were applied every 1.5 min with a 1-second duration (Fig. 1). Within each session, held on a separate day, only one of the two nicotine concentrations was used. Halfway through the experiment (i.e., after 22.5 min), the continuous background air flow into which the olfactometer embedded the short nicotine pulses was switched from clean air to one of the four menthol (diluted in air) conditions:

- Condition 1: clean air was continued without menthol (no menthol/placebo control)
- Condition 2: clean air was replaced by menthol in the air at a concentration of 0.8 μg/mL (menthol-low)
- Condition 3: same as condition 2 except menthol in the air was presented at a concentration of 1.5 μg/mL (menthol-medium)
- Condition 4: same as conditions 2 and 3 except menthol in the air was presented at a concentration of 3.4 μg/mL (menthol-high)

Hence, each of the four menthol conditions was combined with one of the two nicotine concentrations resulting in eight conditions and sessions on eight different days (Fig. 1). All eight conditions were randomized using a Latin square procedure in order to eliminate any carry-over effects. The wash-out period between experiments was at least 3 days.

Participants’ age was restricted to 21–45 years, and they were required to stay within ±20 % of their ideal body weight. Participants were excluded if they had any allergies requiring therapy, chronic or acute infections, had taken any medication within 2 weeks prior to the study or any concomitant medication (except oral contraceptives), had any drug or alcohol abuse problems, gravidity, lactation, relevant loss of blood within 1 month before experiments, any liver or renal diseases, or bronchial asthma.

During initial screening before inclusion into the study, participants’ health was checked by medical history assessment, physical examination, laboratory tests (blood chemistry and hematology, urine analysis, pregnancy test) and measurements of vital signs (blood pressure, pulse, electrocardiogram [ECG]). Patients were excluded if any deviations from normal were identified or if they were pregnant. At the end of the study, participants were examined again using laboratory tests before being discharged. Participants were instructed to abstain from smoking and other trigeminal sensory irritants, such as spicy food and alcoholic beverages, for at least 8 h prior to the study.

In summary, the study was performed following a controlled eightfold, double-blind, cross-over design (blinded for the participants and the scientist who evaluated the data). Twenty healthy adult smokers (smokers on a regular basis smoking at least two non-menthol cigarettes per day; 10 males and 10 females) aged between 21 and 33 years were included. The study was conducted at the Institute of Experimental and Clinical Pharmacology and Toxicology, University Erlangen-Nürnberg, Germany. The protocol was approved by the Institutional Review Board (IRB) of the University, and the study was conducted according to the Declaration of Helsinki on biomedical research involving human subjects (Somerset West amendment 2000). All participants gave their written informed consent prior to their inclusion in the study.

### Statistical analysis

A general linear mixed model for repeated measures was used to fit and analyze the data. Time, treatment (i.e., stimulation with menthol) and treatment by time interaction were used as terms in the model. Participants’ sex was also considered with the aforementioned terms for specific models. Since each subject’s data consisted of correlated longitudinal profiles, the covariance structure that provided the best fit for the data by comparing the associated Akaike’s Information Criterion (AIC) values was identified. The restricted maximum likelihood estimation was used for the linear mixed models. The Tukey–Kramer method for pairwise comparisons of stimulations was used for *P* value adjustments.

Missing data for our analyses were assumed to have gone missing at random. A linear mixed model for repeated-measures analysis of variance (ANOVA) was also used to test for the differences in scores (first half of experiment [pre] minus second half of experiment [during menthol stimulation] between the treatments [i.e., placebo versus menthol stimulation]). In addition, the data are presented as mean estimates with corresponding 95 % confidence intervals for both halves of the experiment.

The association between menthol concentrations (low, medium and high) and each menthol sensation (odor, cooling and pain) was sorted by nicotine stimuli conditions (low nicotine and high nicotine) and assessed using a linear trend analysis. Least-squared means for a factor were obtained assuming that the levels of other factors were equally represented. Statistical significance was evaluated at *P* < 0.05 for all analyses. SAS^®^ (Version 9.1.3) was used to perform the statistical analysis. SAS^®^ Proc Mixed was used for all the analyses, except the trend analysis where the SAS^®^ Proc GLM procedure was used.

## Results

Nineteen of the 20 participants were included in the statistical evaluation. Due to technical problems, VAS data from one individual were lost. Table 1 summarizes the demographic characteristics of the participants, including age, body weight and height, and BMI. No stimulation-related adverse events were observed.

**Table 1:** Descriptive characteristics of the study population

Variable	Male* n* = 10	Female* n* = 10	Overall* n* = 20
Age (years)			
Mean ± SD	27.50 ± 3.24	23.20 ± 1.32	25.35 ± 3.27
(Min–max)	(24–33)	(21–25)	(21–33)
Weight (kg)			
Mean ± SD	71.20 ± 10.89	63.30 ± 7.15	71.20 ± 10.89
(Min–max)	(51–95)	(51–72)	(51–95)
Height (cm)			
Mean ± SD	182.20 ± 8.70	173.20 ± 5.73	177.70 ± 8.53
(Min–max)	(170–200)	(167–183)	(167–200)
BMI (kg m^−2^)			
Mean ± SD	23.90 ± 1.45	21.00 ± 2.67	22.45 ± 2.56
(Min–max)	(21–26)	(16–25)	(16–26)

*SD* standard deviation, *BMI* body mass index

### Psychophysical data

A general linear mixed model for repeated measures was used to fit the psychophysical data. The model terms were time, treatment (i.e., stimulation with menthol) and treatment by time. Since each subject’s data consisted of correlated longitudinal profiles, the covariance structure that provided the best fit for the data by comparing the associated AIC values (i.e., AIC values and −2 log likelihood scores) was the autoregressive covariance structure (first order).

#### Nicotine intensity estimates and potential modulation by menthol

Nicotine stimuli (1-second duration) were clearly perceived by all participants. The time courses of intensity estimates for both painful sensations (burning and stinging) are shown in Fig. 2. Estimated mean values of intensity ratings for the low and high nicotine concentrations across participants are plotted against time, that is, the number of stimuli (interstimulus interval was 1.5 min), separately for both pain sensations (i.e., burning and stinging). In all conditions, an initial increase in intensity estimates of stinging and burning for both nicotine concentrations was followed by a slow decrease in both ratings. This time effect was significant for all conditions during the first half of experiment (effect time ‘pre’; *F* values = 3.13–3.99; *P* value = *P* < 0.0001; Table 2) but not for the second half, indicating that pain perception had reached a steady state. Switching on menthol stimulation in the second half of the experiment did not influence pain estimates of nicotine—neither burning nor stinging pain ratings (see Fig. 2; Table 2).

**Table 2:** Statistical summary for nicotine pain intensity estimates

Nicotine pain sensation	Time effect			Menthol stimulation effect			Interaction time by stimulation		
	*P* value (*F* value)			*P* value (*F* value)			*P* value (*F* value)		
	All	Pre	During	All	Pre	During	All	Pre	During
Stinging (LN)	<0.0001 (3.91)	<0.0001 (3.54)	0.09 (1.56)	0.75 (0.41)	0.70 (0.47)	0.99 (0.04)	0.99 (0.64)	0.96 (0.66)	0.95 (0.67)
Stinging (HN)	<0.0001 (3.41)	<0.0001 (3.13)	0.67 (0.41)	0.85 (0.26)	0.75 (0.40)	0.92 (0.41)	0.38 (1.04)	0.23 (1.16)	0.46 (0.41)
Burning (LN)	<0.0001 (3.01)	<0.0001 (3.33)	0.36 (1.09)	0.95 (0.11)	0.88 (0.22)	0.99 (0.00)	0.82 (0.86)	0.52 (0.97)	0.87 (0.76)
Burning (HN)	<0.0001 (3.35)	<0.0001 (3.99)	0.65 (0.81)	0.21 (1.56)	0.50 (0.80)	0.68 (0.50)	0.95 (0.76)	0.49 (0.99)	0.99 (0.46)

*P* values were derived from a linear mixed model for repeated measures; statistical significance was evaluated at *P* < 0.05. LN, low nicotine; HN, high nicotine

All = whole experimental session (stimulus: 0–30), pre = first half of experiment (stimulus: 1–15) and during = second half of experiment (i.e., during menthol application; stimulus: 16–30)

**Fig. 3:**
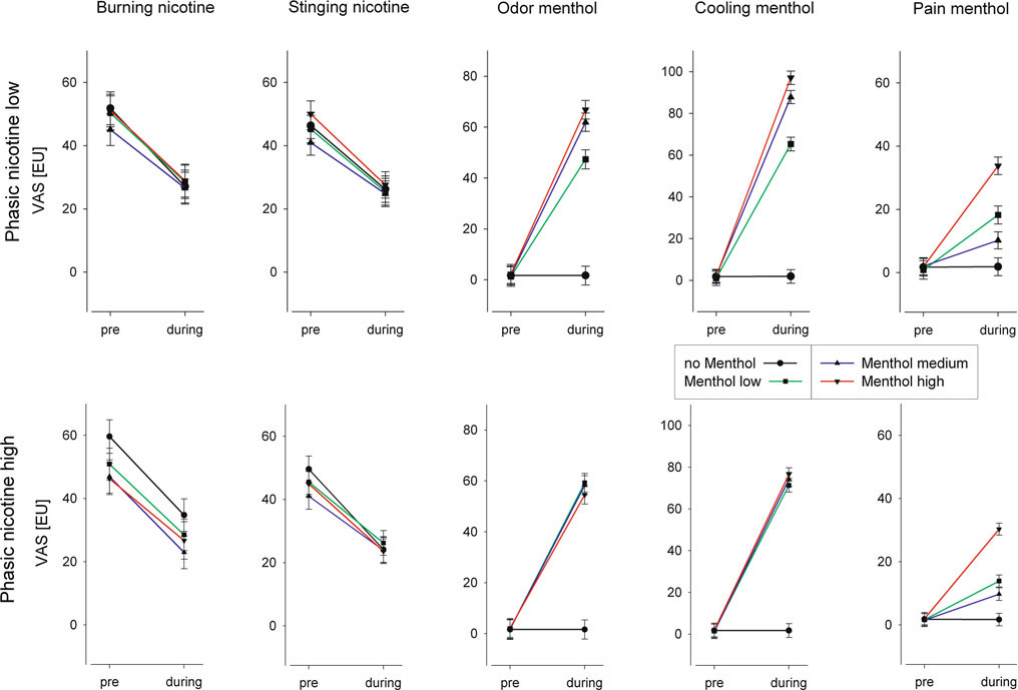
Mean values (and 95 % confidence intervals) of all intensity estimates (19 participants) of the first half of the experiment (pre: before menthol or placebo control was switched on) compared with mean values of all intensity estimates of the second half of the experiment (during: after menthol or placebo control was switched on) for five different sensations measured in eight experimental conditions. Nicotine intensity ratings show a clear decline from ‘pre’ to ‘during’ indicating desensitization in all experimental conditions without the influence of menthol stimulation. Menthol intensity ratings that were close to zero for all three sensations in the first half of the experiment and continued to stay there in the placebo (no-menthol) condition, but increased to different levels after menthol was switched on except for odor and cooling sensations, while high nicotine stimuli were concomitantly presented (VAS = visual analog scale; EU = estimation unit). The estimated mean values were derived from a linear mixed model for repeated measures using a least-square means statement

In Figure 3, the mean intensity estimates for the first half of the experiment (‘pre’ menthol stimulation) are compared with the mean intensity estimates of the second half of the experiment (‘during’ menthol stimulation). Visible differences in baseline (first half of experiment) were accounted for by comparing the mean differences between pre and during menthol stimulation using a general linear model where the term in the model was treatment. This again did not result in any statistically significant effects of menthol on nicotine pain sensations with one exception; nicotine estimates for stinging pain (in the high-nicotine condition) decreased more in the no-menthol condition compared with the menthol-medium condition (see Fig. 3 bottom row second panel from the left). This treatment effect reached the level of significance (mean difference estimates for no menthol versus menthol-medium: 27.4 EU versus 17.8 EU, 95 % CI: 22.79–32.10 versus 13.23–22.28, *P* = 0.0182).

#### Menthol intensity estimates and potential modulation by nicotine

**Fig. 4:**
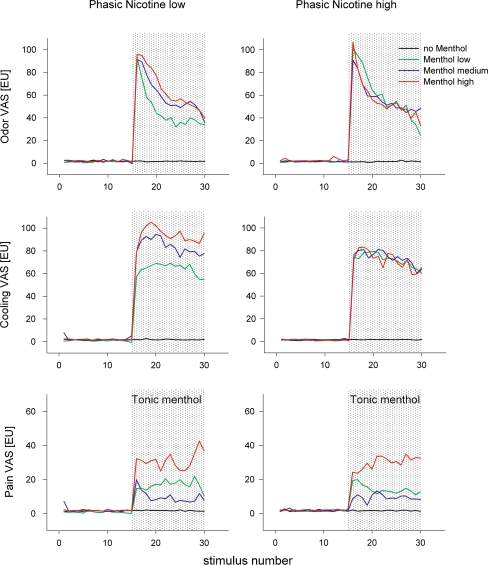
Time course of estimated mean values (19 participants) of odor, cooling and pain intensity estimates for menthol and placebo control. Thirty nicotine stimuli of low (99 ng/mL) and high (134 ng/mL) concentration were presented throughout the entire experimental session. Interstimulus interval: 1.5 min; total duration of experiment: 45 min. During the second half of each experimental session, an additional tonic menthol background stimulus was switched on (*shadowed*) and maintained throughout the rest of the experiment. Three concentrations of menthol (menthol-low: 0.8 μg/mL, menthol-medium: 1.5 μg/mL and menthol-high: 3.4 μg/mL) and a fourth condition with clean air (no menthol) as placebo control were combined with each of the two nicotine concentrations resulting in a total of eight experiments. Participants estimated the smell, cooling and pain sensation elicited by menthol separately on visual analog scales (VAS) after each nicotine stimulus. In the first half of the experiment, all estimates were close to zero. In the second half, estimates remained at this level for placebo control (no menthol), but increased to different levels after menthol was switched on. In the case of the high-nicotine condition, the odor and cooling estimates increased to the same level for all menthol concentrations (EU = estimation unit). The estimated mean values were derived from a linear mixed model for repeated measures using a least-square means statement

Tonic menthol stimulation in the second half of the experiment was clearly perceived by all participants. The concentration of menthol did not change once it was switched on. The time course of intensity estimates for all three sensations elicited by menthol—minty odor, cooling and pain—is shown in Fig. 4. After switching on menthol in the second half of the experiment, an initial increase in odor intensity estimates was followed by a slow and statistically significant decrease in perceived intensity (effect time Table 3; low nicotine (LN): *F* = 6.27, *P* < 0.0001, high nicotine (HN): *F* = 6.49, *P* < 0.0001 and Fig. 4). Cooling estimates did not decrease equally compared with odor intensity estimates, but these changes were also statistically significant with time (effect time Table 3; LN: *F* = 2.44, *P* = 0.002; HN: *F* = 2.34, *P* = 0.004 and Fig. 4). Pain estimates only marginally decreased or even slightly increased, namely for the strong menthol stimulation. These slight changes did not reach a statistical significance (effect time Table 3; LN: *F* = 1.52, *P* = 0.10; HN: *F* = 0.68, *P* = 0.80 and Fig. 4).

**Table 3:** Statistical summary for menthol intensity estimates during menthol application

Menthol sensation	Time effect	Menthol stimulation effect				Interaction time by stimulation
		Overall	Pairwise comparisons			
			ML versus MM	ML versus MH	MM versus MH	
	*P* value (F value)	*P* value (*F* value)				*P* value (*F* value)
Odor (LN)	<0.0001 (6.27)	<0.0001 (14.38)	*P* = 0.22 (*t*_69_ = 1.23)	*P* = 0.09 (*t*_69_ = −1.69)	*P* = 0.62 (*t*_69_ = −0.50)	0.04 (1.42)
Odor (HN)	<0.0001 (6.49)	<0.0001 (10.53)	*P* = 0.86 (*t*_62_ = 0.17)	*P* = 0.80 (*t*_62_ = 0.25)	*P* = 0.94 (*t*_62_ = 0.07)	0.002 (1.79)
Cooling (LN)	0.002 (2.44)	<0.0001 (35.56)	*P* = 0.05 (*t*_69_ = −1.98)	*P* = 0.005 (*t*_69_ = −2.94)	*P* = 0.31 (*t*_69_ = −1.01)	0.26 (1.14)
Cooling (HN)	0.004 (2.34)	<0.0001 (17.62)	*P* = 0.87 (*t*_62_ = 0.17)	*P* = 0.96 (*t*_62_ = 0.05)	*P* = 0.83 (*t*_62_ = 0.22)	0.08 (1.32)
Pain (LN)	0.10 (1.52)	0.005 (4.67)	*P* = 0.40 (*t*_69_ = 0.85)	*P* = 0.09 (*t*_69_ = −1.72)	*P* = 0.01 (*t*_69_ = −2.63)	0.52 (0.97)
Pain (HN)	0.80 (0.68)	0.0003 (7.18)	*P* = 0.45 (*t*_62_ = 0.76)	*P* = 0.014 (*t*_62_ = −2.52)	*P* = 0.002 (*t*_62_ = −3.23)	0.61 (0.93)

*P* values were derived from a linear mixed model for repeated measures; statistical significance was evaluated at *P* < 0.05.* LN*, low nicotine;* HN*, high nicotine

*ML* condition menthol-low, *MM* condition menthol-medium, *MH* condition menthol-high

The significant overall stimulation effect is mainly caused by the menthol placebo condition. The comparisons with the menthol placebo condition are not shown

Interestingly, while a concentration-dependent effect of menthol on all intensity estimates (odor, cooling and pain) was observed (Fig. 4), during stimulation with the lower nicotine concentration, this concentration dependence disappeared for odor and cooling intensity estimates during the higher concomitant nicotine stimulation. In this condition, participants did not seem to be able to discriminate between the different menthol concentrations with respect to odor and cooling. However, with the pain estimates, this surprising influence of nicotine levels on menthol perception was absent. For the pain sensation, participants were still able to recognize the stronger menthol stimuli (that were designed to be painful) and distinguish them from the pain elicited by the medium and low menthol concentrations (that were designed to have virtually no pain sensation). Interestingly, the menthol-medium level seemed to elicit less pain than the menthol-low level. This was supported by the statistical analysis on menthol intensity estimates, which is summarized in Table 3.

Although apparent when visualizing the data (Fig. 4, low-nicotine condition), the discrimination between menthol levels did not reach statistically significant levels for odor intensity estimates in this comparison. However, when evaluating the data using a linear trend analysis, the observed effect became very clear. A statistically significant linear trend for intensity estimates for all three sensations elicited by menthol under low-nicotine conditions (Table 4; odor: *F* = 21.49, *P* < 0.0001; cooling: *F* = 63.91, *P* < 0.0001; pain: *F* = 21.91, *P* < 0.0001) was observed, but under the high-nicotine conditions only the menthol pain intensity estimates showed a statistically significant linear trend (Table 4; odor: *F* = 0.58, *P* = 0.45; cooling: *F* = 0.31, *P* = 0.58; pain: *F* = 50.26, *P* < 0.0001).

**Table 4:** Summary of linear trend analysis for intensity estimates of menthol sensations for both nicotine stimulus conditions by menthol concentrations

Menthol concentration	Low nicotine			High nicotine		
	Odor	Cooling	Pain	Odor	Cooling	Pain
	Mean (95 % CI)	Mean (95 % CI)	Mean (95 % CI)	Mean (95 % CI)	Mean (95 % CI)	Mean (95 % CI)
Low	46.50 (40.73, 52.29)	65.02 (59.83, 70.20)	17.21 (12.82, 21.60)	60.45 (54.11, 66.78)	72.10 (66.61, 77.59)	14.16 (11.09, 17.23)
Medium	60.58 (55.07, 66.09)	84.88 (79.95, 89.81)	9.76 (5.58, 13.94)	57.82 (51.31, 64.32)	74.10 (68.47, 79.72)	9.27 (6.12, 12.41)
High	65.52 (59.91, 71.12)	94.38 (89.37, 99.40)	31.86 (27.56, 36.17)	56.92 (50.40, 63.44)	74.33 (68.69, 79.97)	30.12 (26.93, 33.29)
*P*-trend	*P* < 0.0001 *F* = 21.49	*P* < 0.0001 *F* = 63.91	*P* < 0.0001 *F* = 21.91	*P* = 0.45 *F* = 0.58	*P* = 0.58 *F* = 0.31	*P* < 0.0001 *F* = 50.26

Values shown as least-square mean (95 % confidence intervals). *P* values for trend were derived from a general linear model; statistical significance was evaluated at *P* < 0.05

In Fig. 3, the mean estimates of menthol responses again showed the effect that concomitant nicotine stimuli at the higher concentration resulted in a different cooling perception compared with nicotine stimuli at the lower concentration, that is, taking away the differentiation between different levels of menthol. Menthol’s odor intensity estimates were similar.

#### Sex effects

For the nicotine pain intensity estimates, the effect of sex reached significant levels in the low-nicotine condition for both burning (mean for males: 44.88 EU, 95 %CI: 38.99–50.76; mean for females: 30.5 EU, 95 % CI: 24.85–36.14; *F* = 12.65, *P* = 0.0007) and stinging (mean for males: 38.89 EU, 95 % CI: 35.65–44.13; mean for females: 29.32 EU, 95 % CI: 25.25–33.38; *F* = 13.07, *P* = 0.0006) pain estimates.

For menthol pain intensity estimates, we observed a significant effect of sex (*F* = 5.27, *P* = 0.0247) during the second half of the experiment with higher pain estimates in males (mean 21.24 EU, 95 % CI: 13.35–29.13) compared with females (mean 8.88 EU, 95 % CI: 1.27–16.49) under the low-nicotine condition.

### Acoustic rhinometry

Due to technical reasons, four of 20 participants could not be evaluated statistically for changes in nasal cavity volume. We observed a tendency for reduced nasal volumes in the stimulated left side compared with the non-stimulated right side during the low-nicotine condition in all menthol conditions (effect side of stimulation: *F* = −3.82, *P* = 0.05; volume left versus right: mean change = −0.58 versus 0.06 mL, 95 % CI: −0.98 to −0.17 versus −0.44–0.56 mL). This effect was not observed for the high nicotine concentration (effect side of stimulation: *F* = 0.05, *P* = 0.83).

## Discussion

The purpose of the experiments reported here was to investigate the interactions between two chemical stimuli, menthol and nicotine, both of which activate the olfactory and trigeminal system. More specifically, we wanted to know whether menthol at different levels modulates the perception of the burning and stinging sensations induced by nicotine stimuli. In order to separate the modulatory effects from the direct sensory effects produced by these two compounds, we chose to administer nicotine stimuli phasically, that is, short stimuli repeated every 1.5 min, and the modulatory menthol stimuli tonically. This also helped participants to clearly discriminate between the intensity of the different sensations they had to estimate. In the first half of the experiment, phasic nicotine stimuli were applied alone in order to stabilize participants’ estimations after initial sensitization and desensitization processes. After this, the tonic menthol stimulus at one of the three different concentrations was administered for the entire remainder of the session. Stinging and burning estimates for nicotine significantly decreased during the first half of the experiment reaching the intended stability at perceivable moderate pain levels in the second half with no further significant decrease in pain ratings (Fig. 2, time effect “during” Table 2). Menthol stimulation, at all three concentrations used, did not affect nicotine pain perception, either by reducing or by enhancing intensity estimates for the stinging or burning sensations. There was one isolated exception; nicotine’s stinging pain decreased more in the placebo (no-menthol) condition compared with the menthol-medium condition (see Fig. 3 bottom row second panel from the left). Since there are visible differences in baseline—although not statistically significant—this singular statistical result is probably irrelevant and clearly does not justify a statement that menthol could increase nicotine-induced pain perception.

Surprisingly, nicotine stimuli eliminated the concentration dependence of intensity ratings for menthol’s odor and cooling sensations, but not for pain sensations. However, this modulation was only exerted by high nicotine concentration. In the case of weaker concomitant nicotine stimuli, concentration-dependent ratings could be observed for all intensity estimates of menthol’s odor, cooling and pain sensations. We do not know to what extent the weaker nicotine stimuli might have affected menthol’s perception, because a zero nicotine condition was not included in the study design. As already mentioned, this observation came to our surprise and was not theoretically anticipated.

### Effects of sex, nasal congestion and pharmacology

The study population was balanced for sex but the protocol was not designed to investigate the differences in sex specifically. Nevertheless, we observed lower pain estimates for nicotine- and menthol-induced pain in females compared with males but only in the weaker nicotine stimulation condition. In the literature, examination of differences in sex for pain intensity perception seems to be inconclusive. There are reports of a higher (Cometto-Muniz and Noriega 1985; Shusterman 2002; Olofsson and Nordin 2004), as well as a lower (Nunez et al. 1997; Hashmi and Davis 2009; Breimhorst et al. 2011), pain sensitivity in females. We do not believe that, in this study, the lack of nicotine’s pain modulation by menthol was dependent on this effect of sex, as it was lacking in both conditions, that is, when stimulating with high and low nicotine levels.

Nasal congestion may result in different adaptation processes which may have influenced our data. However, the reported findings were obtained during steady-state nicotine pain perception (second half). Interestingly, there was only a tendency to higher congestion on the stimulated side and only in the case of lower-level nicotine stimulation. Since the lack of pain modulation by menthol was observed with and without congestion, we do not think that congestion is a relevant confounder. Also, we are not aware of a mechanism that would explain the marginal congestion influences on menthol’s odor and cooling perceptions but not on its pain perception (see ‘Nicotine’s action on menthol estimates’).

We cannot rule out the possibility that an unspecific pharmacological effect occurred through the modulation of central nervous system activity by the portion that might have been absorbed through the nasal mucosa, because we did not measure plasma concentrations of nicotine. On the other hand, it could be expected that a more uniform development of the potential unspecific effects from low-to-high nicotine concentrations was similar for all sensory channels, but not the observed switch from discrimination to the lack of discrimination between different stimulus strengths, which, moreover, was dependent on the sensory channel, that is, true for odor and cooling, but not true for pain.

Interestingly, Rosenblatt et al. (1998) found that smokers had elevated thresholds for nicotine stimuli, when sniffed from a vial, compared with non-smokers, but not for menthol. Abstinence from smoking (16–20 h) lowered the threshold but not to the level of non-smokers. Our study population consisted of smokers only, so we conclude that this phenomenon did not influence our data. In addition, we conducted our study following a cross-over design, so any variation in threshold should have been equally distributed across experimental conditions.

### Menthol’s action on nicotine estimates

It is known that menthol activates recombinant mouse TRPA1 channels at low concentrations and inhibits them at higher concentrations; however, this does not seem to be the case for human TRPA1 channels (Xiao et al. 2008). Our human data support this as we do not have any indication that high concentrations of menthol would result in decreased pain perception. Despite the fact that nicotine and menthol both activate TRPA1 channels (Talavera et al. 2009), this potential competition did not result in a modulation of perceived pain intensities in either the sensitizing or the potential inhibitory/desensitizing direction. Levels of nicotine used in this study probably did not reach concentrations in the mucosa that are required to activate TRPA1 channels (Talavera et al. 2009), eliminating the potential for interaction. Hence, the perceived burning and stinging sensations of nicotine stimuli most likely originated from the activation of nAChRs (Thuerauf et al. 2006). So far, there are no published data on a potential interaction of menthol with nicotine at the nAChR. Due to the lack of evidence from our psychophysical data, there is no reason to postulate such an interaction.

### Nicotine’s action on menthol estimates

Although this study was not designed to investigate the effects of nicotine stimuli on the perception of menthol stimulation, the effect of eliminating participants’ discrimination of different menthol levels for odor and cooling by the strong nicotine stimuli seemed to be robust. The question about the underlying mechanisms, that is, where in the information processing chain did this happen and how, cannot be answered on the basis of these data. However, since the effect occurred in two different sensory systems, there is room for some speculation. The loss of discrimination of menthol’s different concentrations was observed in the olfactory and TRPM8-related somatosensory system but not in the TRPA1- and nAChR-related nociceptive system (see above). Interactions of this kind could chiefly take place in the periphery or central nervous system.

For the periphery, there is the possibility that nicotine exerts a, to date, unknown modulation of (a) olfactory receptors that are sensitive to menthol and (b) of TRPM8 channels. Both modulations require a higher rather than a lower level of nicotine. A possible explanation for the lack of an effect on pain is that (c) nicotine, at the concentrations used in this experiment, is not a competitor of menthol at the TRPA1 channels, so the nicotine stimuli at the levels used only activated nAChRs.

In a recent study in mice using plethysmography as a surrogate for airway irritation, menthol had an inhibitory effect on acrolein, acetic acid and cyclohexanone-induced nociception (Willis et al. 2011). Since all these compounds activate TRPA1 channels and we believe that the nicotine concentrations in our study were not high enough to stimulate TRPA1 channels, these results do not help us to interpret our data nor do they contradict our findings.

Next to potential peripheral interactions at the receptor level, there are possibilities for interactions across sensory channels based on axon reflexes as well (Bayliss 1901; Silver and Finger 2009). Indeed, studies in rats have demonstrated that some trigeminal ganglion cells with sensory endings in the nasal epithelium also have branches reaching directly into the olfactory bulb and even into the spinal trigeminal complex (Schaefer et al. 2002). These unique morphological structures could be the substrate for the modulation of incoming sensory information with respect to smell, pain, temperature and touch. It is thought that axon reflexes, initiated where collaterals branch off the afferent nerve, could modify the sensitivity of peripheral receptive structures by the release of peptides such as substance P and calcitonin gene–related peptide (CGRP) in the tissue innervated by these collaterals. Indeed, it has been found that electrical stimulation of the ethmoidal nerve inhibits olfactory bulb activity in cats and rabbits (Kerr and Hagbarth 1955; Stone et al. 1968). In Andre Holley’s laboratory, stimulation of the trigeminal nerve was also found to inhibit olfactory receptor cell activity (Bouvet et al. 1987). In humans, there are a number of reports of interactions between the somatosensory and olfactory systems that seem to be inhibitory if both stimuli were applied in a close temporal context (Cain and Murphy 1980; Brand 2006). In our laboratory, we tried to find interactions between these two systems in humans by electrical stimulation of the facial skin on the perception of olfactory stimuli, but were unsuccessful (Livermore et al. 1993). For the results described in this paper, we do not think that they fit into an axon reflex type of mechanism. Our data reveal a dose-dependent interaction between nicotine stimuli and menthol’s odor and cooling intensity perceptions. This interaction is specific, because menthol’s pain intensity perception is excluded, although the nociceptive neuronal activity should pass through the same spinal trigeminal complex as the cooling information. Also, there is not just inhibition as one might expect from previous work, but a differentiated influence: (1) increase in the intensity estimates for the weaker menthol stimulus (on odor and cooling), (2) no effect on the estimates for the menthol-medium stimulus and (3) a reduction in intensity estimates for the strong menthol stimulus (on odor and cooling). To our knowledge, there is no mechanism based on axon reflexes that could explain these divergent phenomena. Hence, we favor a more central location of the observed interactions.

For the central nervous system, one could assume that information about noxious stimuli is the most relevant input for the organism so that, at more intense pain perception, the smell or cooling information is less relevant for the integrity of the organism and therefore while still perceived, will not be differentiated. In the case of lower pain perception from the less concentrated nicotine stimuli, the discrimination between different intensities of cooling and odor is retained, because of their relatively higher relevance. This would represent a modulation of selective attention, which has been shown to affect pain perception (Marchand and Arsenault 2002; Villemure and Bushnell 2002) but not, to our knowledge, cooling and odor perception by competing pain. However, a study where participants were exposed to visual and noxious heat stimuli found that attention was preferentially shifted to the painful stimulus (Miron et al. 1989), which supports our interpretation that, in case of competing sensory information, pain processing wins (Bain 1868). However, both hypotheses—the peripheral and the central—warrant further investigations. In order to determine whether the observed effects are peripheral or central, it would be advisable to conduct a study in which recordings are obtained from peripheral sensory structures. Fortunately, such recording techniques are available: The electro-olfactogram (EOG) is a summated generator potential of olfactory receptor cells (Kobal 1981; Hummel et al. 1996) and therefore can be used to demonstrate peripheral olfactory effects. The negative mucosa potential (NMP) that correlates with somatosensory activities is a peripheral response as well (Kobal 1985; Thurauf et al. 1993). We plan to further analyze the observed phenomena by using both recording techniques combined with chemosensory evoked potentials (Lötsch et al. 1997; Knecht and Hummel 2004). A zero nicotine condition needs to be included as well in order to show the total modulatory effect of nicotine on menthol intensity perception.

In summary, results from this study demonstrate that stinging and burning intensity estimates for repeated phasic nicotine stimuli significantly decreased during the first half of the experiment. Additional continuous menthol stimulation did not alter the nicotine-induced steady-state pain sensations. Surprisingly, there was a nicotine effect on the menthol odor and cooling sensations, indicating potential modality-specific interactions at peripheral receptors or selective attention-related interactions at higher levels in the central nervous system.
